# Highlights from the first interdisciplinary summit of the European Association of Cardiovascular Imaging and the European Society for Cardiovascular Radiology

**DOI:** 10.1093/ehjci/jeag031

**Published:** 2026-04-14

**Authors:** Rozemarijn Vliegenthart, Anna Baritussio, Ricardo P J Budde, Gianluca Pontone, Marly van Assen, Jean-Nicolas Dacher, Ibrahim Danad, Victoria Delgado, Marc R Dweck, Pim van der Harst, Alexander Hirsch, Merel Huisman, Sebastian Kozerke, Théo Pezel, Francesca Pugliese, Mark Westwood, Michelle C Williams, Maja Hrabak Paar, Robert Manka, Rodrigo Salgado, Robin Nijveldt

**Affiliations:** Department of Radiology, University Medical Center Groningen, Groningen, the Netherlands; Department of Cardiac, Thoracic, Vascular Sciences and Public Health, Padua University Hospital, Padua, Italy; Department of Radiology & Nuclear Medicine, Erasmus Medical Center, Rotterdam, the Netherlands; Department of Perioperative Cardiology and Cardiovascular Imaging, Centro Cardiologico Monzino IRCCS, Milan, Italy; Department of Biomedical, Surgical and Dental Sciences, University of Milan, Milan, Italy; Department of Radiology and Imaging Sciences, Emory University School of Medicine, Atlanta, GA, USA; Department of Radiology (Cardiac Imaging Unit), CHU et Université de Rouen-Normandie, Rouen, France; MIRACL.ai Laboratory, Multimodality Imaging for Research and Analysis Core Laboratory and Artificial Intelligence, University Hospital of Lariboisiere (AP-HP), Paris, France; Department of Cardiology, Radboud University Medical Center, Geert Grooteplein 10, 6525 GA Nijmegen, the Netherlands; Department of Cardiology, Hospital Universitari Germans Trias i Pujol, Badalona, Spain; British Heart Foundation Centre for Research Excellence, University of Edinburgh, Edinburgh, UK; Department of Cardiology, University Medical Center Utrecht, Utrecht, the Netherlands; Department of Cardiology, Erasmus Medical Center, Rotterdam, the Netherlands; Department of Radiology and Nuclear Medicine, Radboud University Medical Center, Nijmegen, the Netherlands; Institute for Biomedical Engineering, University and ETH Zurich, Zurich, Switzerland; MIRACL.ai Laboratory, Multimodality Imaging for Research and Analysis Core Laboratory and Artificial Intelligence, University Hospital of Lariboisiere (AP-HP), Paris, France; Departments of Cardiology and Radiology, University Hospital of Lariboisiere, (Assistance Publique des Hôpitaux de Paris, AP-HP), Paris, France; Université Paris Cité, Inserm MASCOT—UMRS 942, Paris, France; Department of Radiology, Queen Mary University of London, London, UK; Department of Cardiology, Barts Heart Centre, Barts Health NHS Trust, London, UK; British Heart Foundation Centre for Research Excellence, University of Edinburgh, Edinburgh, UK; Department of Radiology, University Hospital Center Zagreb, Zagreb, Croatia; Department of Cardiology, University Hospital Zurich, Zurich, Switzerland; Department of Radiology, Antwerp University Hospital, Antwerp, Belgium; Department of Cardiology, Radboud University Medical Center, Geert Grooteplein 10, 6525 GA Nijmegen, the Netherlands

## Introduction

On 12 April 2025, the European Association of Cardiovascular Imaging (EACVI) and the European Society of Cardiovascular Radiology (ESCR) hosted the *Hot Topics in Cardiac Imaging Summit* at Amsterdam UMC in the Netherlands. This event followed the successful Global CMR2024 meeting and was designed to deepen collaboration between cardiologists and radiologists. Over 100 participants gathered for scientific sessions, industry updates, and panel discussions, with a programme focused on four pressing themes: non-acute coronary artery disease (CAD), sustainability in imaging, valvular heart disease (VHD), and artificial intelligence (AI).

## Session I: imaging in suspected, non-acute CAD

**Figure 1:**
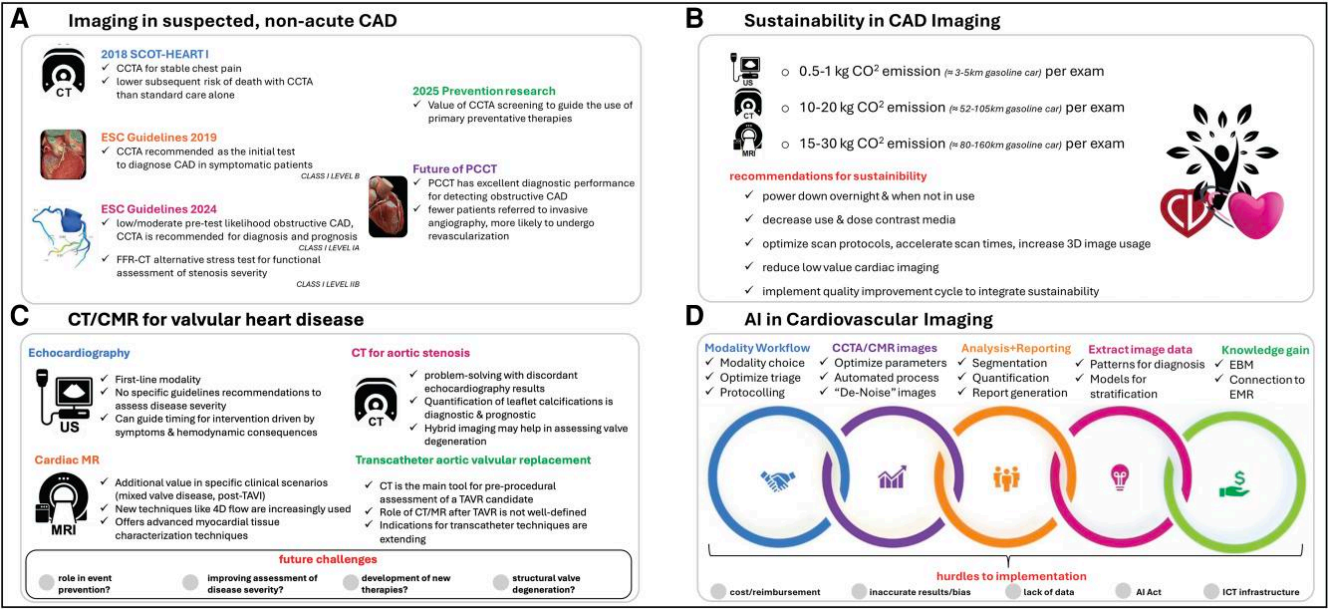
Overview of the main points of the hot topic sessions.

The day opened with a discussion on one of the most common challenges in cardiology practice: how best to evaluate patients with non-acute chest pain (*Figure 1A*). Current ESC guidelines introduce a more comprehensive definition of CAD, including structural and functional changes in epicardial vessels and the microcirculation.^1^ It emphasizes determining pre-test probability (PTP), yet in practice, PTP is often skipped, and existing calculators still tend to overestimate the prevalence of obstructive CAD.

Attention then turned to coronary CT angiography (CCTA), which has become firmly established as the first-line test for most patients. With its very high negative predictive value, CCTA offers reassurance in ruling out obstructive CAD and, as seen in large trials, helps reduce rates of myocardial infarction through preventive therapy.^2^ Careful patient preparation—controlling heart rate and administering nitroglycerin before the scan—remains crucial to achieving diagnostic image quality. Absolute contraindications are limited to severe contrast allergy and the inability to comply with instructions.

For patients at higher risk, with known CAD and/or after intervention, functional imaging continues to play an important role. Positron-emission tomography perfusion stands out for accuracy, although its use is limited by availability and cost. Computed tomography (CT)-derived fractional flow reserve shows promise but is restricted to epicardial vessels. Hybrid strategies combining multiple tests are often more complex than beneficial. The key message: avoid stacking modalities and instead match the test to the patient’s risk and clinical profile.

New technology is also reshaping the field. Photon-counting CT (PCCT), introduced into clinical practice just a few years ago, brings higher spatial resolution and improved spectral imaging.^3^ This reduces artefacts such as calcium blooming, offers clearer plaque characterization, and may prevent unnecessary referrals for invasive angiography. While still limited in availability, PCCT has the potential to refine diagnostic accuracy and expand CT’s role in CAD assessment.

The consensus from the session was clear: non-invasive imaging is central to managing suspected CAD. CCTA is the preferred first test in most cases, functional imaging remains essential in selected patients, and PCCT represents the next leap forward.

## Session II: sustainability in CAD imaging

From there, the conversation shifted to a different kind of challenge: how to balance diagnostic excellence with environmental responsibility. Cardiac imaging is energy-intensive, and the healthcare sector overall contributes significantly to global carbon emissions. This session asked whether imaging can be streamlined to serve both patients and the planet (*Figure 1B*).

Cardiovascular magnetic resonance imaging (CMR) protocols were the first focus. Over the years, scan times have grown longer and data volumes larger, but the incremental information gained has not always matched this expansion. Advances in undersampling allow acceleration of scans by a factor of two to five without compromising diagnostic yield, and AI can further denoise and sharpen reconstructions. New approaches, such as contrast-free viability imaging, offer hope of reducing reliance on gadolinium, although more validation is needed. The message was that shorter, smarter CMR protocols are possible—and necessary.

Attention then turned to CT. The demand for CCTA has surged since its elevation to a class I recommendation in ESC guidelines. Streamlining workflows could help manage this load: for example, moving patient preparation outside the scanner room, and omitting calcium scoring in younger patients. While virtual non-contrast images from spectral CT are being explored, they currently underestimate calcium burden and cannot fully replace a true calcium score. Conversely, in low-risk patient groups, a calcium score of zero can help avoid unnecessary CCTA, provided clinicians remain cautious about younger individuals who may still harbour obstructive CAD.

Finally, the broader ecological impact was considered. Among radiological imaging modalities, magnetic resonance imaging consumes the most energy, followed by CT and ultrasound.^4^ Nuclear medicine brings additional challenges with radioactive waste. Scanners consume large amounts of energy even when idle, raising the question of whether they could be powered down during unused hours. Contrast media and data storage further contribute to the environmental footprint. Solutions include developing leaner protocols, limiting unnecessary follow-up scans, raising awareness among both clinicians and patients, and pushing vendors towards more efficient hardware and software.

The discussion concluded with a call to action: cardiac imagers must assume responsibility for sustainability, tailoring protocols carefully, limiting waste, and partnering across disciplines to promote greener imaging practices.

## Session III: CT and CMR for valvular heart disease

The next session explored how modern imaging can best support patients with VHD. Echocardiography remains the cornerstone, but complex cases increasingly call for complementary tools (*Figure 1C*).

Current guidelines recommend echocardiography as the primary imaging technique to assess VHD severity, although this can be challenging in some clinical situations.^5^ Guidelines also emphasize the integral role of multimodality imaging. There is no single gold standard, and the choice of modality often depends on the clinical context. Several important questions remain unanswered: Should we intervene earlier in asymptomatic patients with severe VHD? How should we monitor asymptomatic patients? Could advanced imaging parameters, such as myocardial strain or tissue characterization, enhance the timing of interventions?

CMR is particularly valuable in VHD. It enables detailed assessment in mitral and aortic regurgitation, helps evaluate prosthetic valves, and plays a central role in congenital heart disease follow-up, particularly for the pulmonary valve. Technologies like 4D flow allow comprehensive mapping of blood movement through the heart, while tissue characterization techniques add insight into how valves affect the myocardium.

CT, meanwhile, has transformed the evaluation of aortic stenosis. Discordant results between echo and clinical presentation are common, especially in low-flow, low-gradient cases. Quantitative CT calcium scoring provides an objective measure that strongly correlates with outcomes and is now incorporated into ESC guidelines.^5^ New developments, such as integrating fibrotic and calcific components into a ‘fibrocalcific score’,^6^ promise even more refined prognostication, although they are not yet in routine use.

As for interventions, the rise of transcatheter techniques—especially transcatheter aortic valve implantation—has been remarkable. CT is indispensable for pre-procedural planning, helping determine valve size and anatomy, while CMR provides complementary information in select cases.^7^ Echocardiography remains the primary modality for follow-up, with CT and CMR playing little role after the procedure.

The overarching lesson was that multimodality imaging strengthens VHD management, but clearer guidance is needed on which tools to apply at each stage of the disease, particularly as transcatheter options expand.

## Session IV: artificial intelligence in cardiovascular imaging:

The final session addressed a rapidly evolving frontier: AI (*Figure 1D*). AI is reshaping how cardiac imaging is performed, interpreted, and even conceptualized, but challenges remain in ensuring responsible adoption.^8^

In CT, AI is increasingly embedded across the workflow—from image acquisition to plaque analysis. Automated software can quantify coronary plaque burden, model fractional flow reserve, and support decision-making, potentially reducing unnecessary angiography and aiding revascularization planning. Machine-learning models combining imaging with clinical data hold promise for more personalized risk prediction, identifying patterns invisible to traditional statistics.

In CMR, AI is transforming speed and reproducibility. AI-assisted protocol optimization could shorten scan times while automated segmentation improves consistency in measuring cardiac structure and function. Machine-learning models are also being developed to cluster patients into phenotypic subgroups, which may improve prognostication and enable more targeted therapies. Early evidence suggests that integrating CMR with CT data may further enhance predictive power.^9^ Yet, clinicians must be able to critically appraise AI outputs and avoid automation bias. Transparency, in the form of explainability, is thought to help build trust and improve usability.

Beyond technology, regulatory and ethical considerations are important. AI in imaging is recognized as a medical device under the MDR. Since healthcare is classified as high-risk under the European AI Act, it mandates compliance with requirements on safety, transparency, and human oversight.^10^ The European Health Data Space will establish a secure and interoperable EU-wide framework for the primary and secondary use of health data, the latter facilitating AI development. Still, clinical validation remains a bottleneck; few tools have yet proven they improve outcomes, reduce costs, or save time at scale. Risks include overreliance on false positives, bias in training data, and security concerns, particularly around large language models used for reporting.

The consensus was optimistic but cautious. AI has immense potential to improve workflow, diagnosis, and prognosis in cardiovascular imaging, but it must be deployed responsibly. Progress will depend on building well-annotated datasets, rigorous validation, IT infrastructure, regulatory oversight, and clinician trust.

## Conclusion

The *Hot Topics in Cardiac Imaging Summit 2025* showcased both the progress and the challenges facing cardiovascular imaging today. Across all sessions, one theme stood out: collaboration—between cardiology and radiology, between technology and clinical practice, and between innovation and sustainability. Non-invasive imaging continues to evolve, with CCTA central in CAD, PCCT on the horizon, and multimodality approaches refining VHD assessment. Sustainability must now become a guiding principle, pushing for shorter, smarter, and greener imaging practices. AI offers transformative possibilities but demands careful regulation, transparency, and validation. Above all, the meeting reinforced that progress in cardiovascular imaging will come from working together. By combining expertise, respecting complementary skills, and addressing the broader responsibilities of healthcare, the community can ensure that innovation translates into better patient outcomes and a more sustainable future.

## Data Availability

No data were generated or analysed for or in support of this paper.

## Appendix 1

*EACVI-ESCR Summit organizing committee*

Anna Baritussio (EACVI Councilor CMR 2022–2026), Ricardo Budde (ESCR Secretary), Robert Manka (EACVI Vice-president-elect CMR 2024–2026), Robin Nijveldt (EACVI Vice-president CMR 2022–2024), Maja Hrabak Paar (ESCR Chair Educational Committee), Gianluca Pontone (EACVI Vice-president Nuclear Cardiology and CCT 2022–2024), Rodrigo Salgado (ESCR President 2024–2026), Rozemarijn Vliegenthart (ESCR President 2022–2024).
